# Relation Between Body Composition, Systemic Inflammatory Response, and Clinical Outcomes in Patients Admitted to an Urban Teaching Hospital with COVID-19

**DOI:** 10.1093/jn/nxab142

**Published:** 2021-06-03

**Authors:** Josh McGovern, Ross Dolan, Conor Richards, Barry J Laird, Donald C McMillan, Donogh Maguire

**Affiliations:** Academic Unit of Surgery, School of Medicine, University of Glasgow, New Lister Building, Royal Infirmary, Glasgow, United Kingdom; Academic Unit of Surgery, School of Medicine, University of Glasgow, New Lister Building, Royal Infirmary, Glasgow, United Kingdom; School of Medicine, University of Glasgow, Glasgow, United Kingdom; Institute of Genetics and Molecular Medicine, University of Edinburgh, Edinburgh, United Kingdom; Academic Unit of Surgery, School of Medicine, University of Glasgow, New Lister Building, Royal Infirmary, Glasgow, United Kingdom; Emergency Department, Glasgow Royal Infirmary, Glasgow, United Kingdom

**Keywords:** body composition, obesity, sarcopenia, CT, COVID-19

## Abstract

**Background:**

COVID-19 has been associated with cases of severe respiratory illness, admissions to intensive therapy units (ITUs), and high mortality rates.

**Objectives:**

The aim of the present study was to examine the relation between computed tomography- body composition (CT-BC) measurements, systemic inflammation, and clinical outcomes in those with COVID-19.

**Methods:**

Patients who presented to our institution between March 17 and May 1, 2020, with a positive PCR test for COVID-19 or characteristic radiological changes, were assessed for inclusion. Data collected included general demographic details, clinicopathological variables, poGPS, NLR , CT-BC measurements, and clinical outcomes including ITU admission and 30-d mortality, of those admitted.

**Results:**

Sixty-three patients met the study inclusion criteria. Forty-two patients (67%) were aged ≥70 y, 30 (47.6%) were male and 34.9% ( *n* = 22) had a poGPS ≥1. ITU admission was significantly associated with a high VFA ( *P* < 0.05). Thirty-day mortality was associated with high VFA (*P* < 0.05) and low SMI (*P* < 0.05).

**Conclusions:**

Sarcopenia in the presence of obesity was associated with clinical outcomes including greater 30-d mortality.

## Introduction

The WHO declared the outbreak of novel coronavirus 19 (COVID-19) a global pandemic on March 11, 2020 (1). Despite an expansion in resources for testing and contact tracing, hospital admissions and death rates within the United Kingdom remained high (2). Since first identified, COVID-19 has been associated with cases of severe respiratory illness, often requiring hospitalization and in some cases admission to an intensive therapy unit (ITU), as well as high mortality rates (3). With the potential for health services to become overwhelmed due to finite resources such as ventilators and level 3 ITU beds available and staffed, factors that aid in prognostication are essential to triage those admitted with COVID-19. This could provide an invaluable insight in the fight against the current global pandemic.

A marked systemic inflammatory response has been identified as one of the signs of severe COVID-19 (4). Recent studies have shown that severe systemic inflammation is associated with mortality in those with COVID-19, suggesting that it can have a role in determining prognosis. Furthermore, obesity, as measured by BMI (5) and visceral fat area (VFA) (6, 7) derived from computed tomography (CT) image analysis, has been reported to have a detrimental impact on clinical outcomes in those with COVID-19. The relation between CT-derived measures of body composition including low skeletal muscle mass and density, systemic inflammation, and outcomes in those with cancer have previously been reported (8, 9). However, to date, there have been no studies exploring the relation between systemic inflammation, CT-derived body composition (CT-BC) measurements, and clinical outcomes in those with COVID-19.

Therefore, the aim of the present study was to examine the relation between CT-BC measurements, systemic inflammatory status, and clinical outcomes in those with COVID-19.

## Methods

Data were collected on patients who attended the Emergency Department (ED) and Acute Assessment Unit (AAU) at Glasgow Royal Infirmary (GRI), Glasgow, United Kingdom, during the initial 7-wk period of the COVID-19 pandemic in Glasgow city (March 17, 2020 to May 1, 2020). GRI is a university teaching hospital, serving an urban population with a high burden of socioeconomic deprivation. In line with UK National Health Service (NHS) policy, this study was approved by the NHS Greater Glasgow and Clyde Caldicott guardian. The study protocol (GN20AE307) was approved by the North West England—Preston research ethics committee (20/NW/0336) and registered with clinicaltrials.gov (NCT04484545).

**FIGURE 1:**
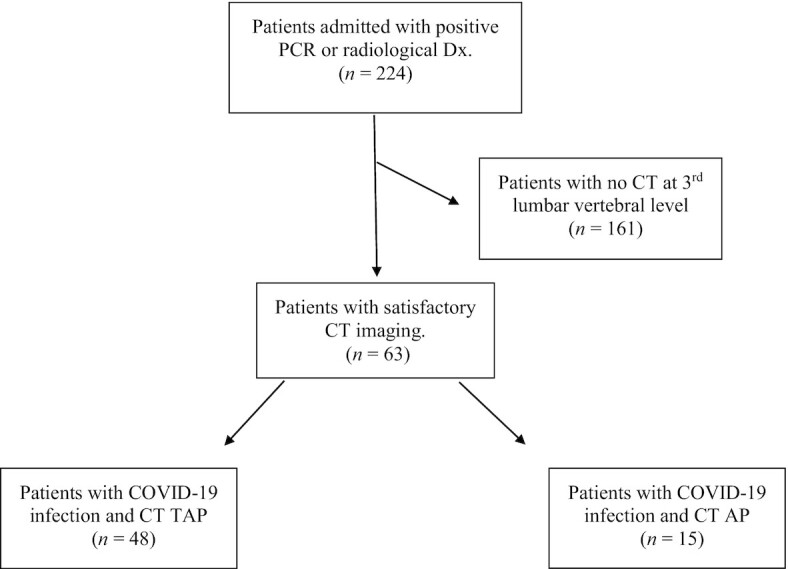
Flow diagram of included patients with COVID-19 and satisfactory CT imaging. AP, abdomen and pelvis; COVID-19, coronavirus disease; CT, computed tomography; Dx, diagnosis; TAP, thorax, abdomen, and pelvis.

Patients displaying clinical signs or symptoms consistent with possible COVID-19 (as defined by Health Protection Scotland) (10), at the time of presentation to the ED and AAU, were assessed for inclusion in the study. Patients were then further analyzed to identify those with either a positive PCR test or radiological changes characteristic of severe acute respiratory syndrome coronavirus 2 (SARS-CoV-2), reported on chest X-ray or CT thorax, by a board-certified radiologist. Finally, patients with confirmed COVID-19 were then assessed to identify those who had CT imaging within 3 mo of the diagnosis (see **Figure 1**). Eligible CT imaging required cross-sectional scanning at the level of the third lumbar vertebra. Patients whose scans were taken outwith this period were excluded from the study. Furthermore, scans with significant movement artefact or missing region of interest were not considered for inclusion.

Routine demographic details, clinical observations, hematological and biochemical laboratory results, as well as clinical outcome data were recorded. Age, sex, ethnicity, BMI, and diagnostic modality confirming COVID-19 as well as date of diagnosis were minimal inclusion criteria. Age categories were grouped to <70 y or ≥70 y. Social deprivation was defined by the Scottish Indices of Multiple Deprivation 2019 based on individuals’ home postcodes. Ethnicity was classified as white or other ethnic group. Admission serum C-reactive protein (CRP), albumin, and differential blood cell counts were categorized using local reference intervals. Neutrophil:lymphocyte ratio (NLR) (11) and the perioperative Glasgow Prognostic Score (poGPS) (12) were used to assess systemic inflammation. For this study, thresholds of NLR <3, 3–5, >5 were chosen and categorized as “mild,” “moderate,” and “severe” systemic inflammatory response, respectively. poGPS values were grouped into “noninflamed” (i.e., poGPS = 0) and “inflamed” (i.e., poGPS = 1 or 2) cohorts. Primary outcomes measured were intensive care admission and mortality within 30 d of diagnosis with COVID-19.

### Body composition analysis

Each CT image was individually analyzed using ImageJ—a free to download, Java-based program developed by NIH (NIH ImageJ version 1.47; http://rsbweb.nih.gov/ij/) shown to provide reliable measurements (13). Body composition measurements derived from the CT image slice at L3 included total fat area (TFA), visceral fat area (VFA), and skeletal muscle area (SMA). Attenuation thresholds were from −190 to +30 Hounsfield units (HU) for fat and −29 to +150 HU for muscle. The TFA was quantified by depicting the outer contours of the abdominal wall, compared with the inner contour of the psoas and abdominal wall muscles for VFA. Similarly, SMA was measured by manually delineating muscle areas including the quadratus lumborum, psoas, rectus abdominus, and erector spinae muscles, and the internal transverse and external oblique muscle groups. Skeletal muscle radiodensity (SMD) was calculated (in Hounsfield units) as the mean of the measured muscle area used to calculate SMI. Subcutaneous fat area (SFA) was calculated by subtraction of the VFA from TFA. SFA and SMA measurements were then normalized by division of the patient's height in meters squared to generate a subcutaneous fat index (SFI: centimeters squared/meters squared) and skeletal muscle index (SMI: centimeters squared/meters squared). These indices were then compared with established thresholds for body composition status (see **Table 1**).

### Statistical analysis

Demographic data, CT-BC measurements, poGPS, and NLR were presented as categorical variables. Categorical variables were analyzed using χ^2^ test for linear-by-linear association.

Missing data were excluded from analysis on a variable-by-variable basis. Two-tailed *P* values <0.05 were considered statistically significant. Statistical analysis was performed using SPSS software version 25.0 (SPSS Inc.).

## Results

Of the 224 patients admitted to GRI during the study period, 63 met the study inclusion criteria. The clinicopathological characteristics at presentation are shown in **Table 2**. Forty-two patients (67%) were aged ≥70 y. Thirty (48%) participants were male. The majority of patients were of white, Scottish ethnicity (94%). With the exception of hypertension, which was present in 34 (53%) individuals included, the majority of patients had no history of comorbid disease—heart failure (13%), type 2 diabetes (28%), liver disease (10%), chronic renal failure (18%), asthma (21%), and chronic obstructive pulmonary disease (22%). Of those included, 11 (18%) patients had active cancer. Of those admitted, 16% (*n* = 10) were current smokers, with 28 (44%) patients reporting a past history of smoking.

**TABLE 1:** Results of body composition analysis of patients with COVID-19 determined from CT^1^

Body composition measurement	Frequency, *n* (%)
*Obesity*	
High SFI (14): males >50.0 cm^2^/m^2^; females >42.0 cm^2^/m^2^	No: 16 (25.4); yes: 47 (74.6)
Visceral obesity (15, 16): VFA: males >160 cm^2^; females >80 cm^2^	No: 21 (33.3); yes: 42 (66.7)
*Sarcopenia*	
SMI (15):	
Males: BMI <25 kg/m^2^ and SMI <43 cm^2^/m^2^, or BMI ≥25 and SMI <53 cm^2^/m^2^ Females: BMI <25 and SMI <41 cm^2^/m^2^, or BMI ≥25 and SMI <41 cm^2^/m^2^	No: 24 (38.1); yes: 39 (61.9)
*Myosteatosis*	
SMD (15): BMI <25 and SMD <41 HU, or BMI ≥25 and SMD <33 HU	No: 12 (19.0); yes: 51 (81.0)

^1^COVID-19, coronavirus disease; CT, computed tomography; HU, Hounsfield units; SFI, subcutaneous fat index; SMD, skeletal muscle radiodensity; SMI, skeletal muscle index; VFA, visceral fat area.

**TABLE 2:** Patient characteristics^1^

Demographics	Frequency, *n* (%)
Sex	
Male	30 (47.6)
Female	33 (52.4)
Age, y	
<70	21 (33.3)
≥70	42 (66.7)
Ethnicity	
White	59 (93.7)
Other	4 (6.3)
BMI, kg/m^2^	
≥25	31 (49.2)
≥30	15 (23.8)
Smoking status	
Current	10 (15.9)
Ex	28 (44.4)
Never	25 (39.7)
Alcohol excess history	
Yes	11 (17.5)
No	52 (82.5)
Clinical frailty	
Yes	45 (71.4)
No	16 (25.4)
Not recorded	2 (3.2)
Comorbidities	
Liver disease	
Yes	6 (9.5)
No	57 (90.5)
Hypertension	
Yes	34 (53.1)
No	29 (45.3)
Heart failure	
Yes	8 (12.7)
No	55 (87.3)
T2DM	
Yes	18 (28.1)
No	45 (70.3)
Chronic renal failure	
Yes	11 (17.5)
No	52 (82.5)
Asthma	
Yes	13 (20.6)
No	50 (79.4)
COPD	
Yes	14 (22.2)
No	49 (77.8)
Active cancer	
Yes	11 (17.5)
No	52 (82.5)
CT imaging	
Thorax, abdomen, and pelvis	48 (76.2)
Abdomen and pelvis only	15 (23.8)
Inflammatory status	
CRP, mg/L	
≥10	52 (82.5)
≥80	31 (49.2)
≥150	14 (22.2)
Albumin, g/L	
<25	13 (20.6)
≥25	50 (79.4)
NLR	
<3	10 (15.6)
3–5	12 (18.8)
>5	41 (64.1)
poGPS	
0	41 (65.1)
1–2	22 (34.9)
Primary outcomes	
ITU admission	
Yes	3 (4.8)
No	60 (95.2)
30-d mortality	
Yes	11 (17.5)
No	52 (82.5)

^1^COPD, chronic obstructive pulmonary disease; CRP, C-reactive protein; CT, computed tomography; ITU, intensive therapy or care unit; NLR, neutrophil:lymphocyte ratio; poGPS, perioperative Glasgow Prognostic Score; T2DM, type 2 diabetes mellitus.

The median BMI was 26.5 kg/m^2^, with 49% (*n* = 31) of patients having a BMI ≥25, and 24% (*n* = 15) having a BMI ≥30. A severe systemic inflammatory response (CRP ≥80 g/L) was present in almost half of individuals studied (49%) (*n* = 31), and a very severe systemic inflammatory response (CRP ≥150 g/L) was present in 14 (22%). A serum albumin <35 mg/L was present in 84% (*n* = 53) of individuals. Seventeen (27%) patients had a poGPS score of 1, and 5 (8%) had a poGPS of 2. An NLR of 3–5 was reported in 22% (*n* = 14) of individuals studied, with 39 (62%) having an NLR >5, indicating moderate and severe inflammation, respectively.

Of the patients with imaging deemed to be of sufficient, analyzable standard for inclusion within the study, 48 (76%) had a CT thorax, abdomen, and pelvis, with 24% (*n* = 13) having a CT abdomen and pelvis only. CT-BC measurements included were VFA, SFI, SMI, and SMD using predefined thresholds. CT-BC analysis results are shown in Table 1. A high VFA was present in 67% (*n* = 42) of patients. VFA was significantly associated with BMI (*P* < 0.01), smoking status (*P* < 0.01), active cancer (*P* < 0.01), ITU admission (*P* < 0.05), and 30-d mortality (*P* < 0.01; **Table 3**). A high SFI was present in a greater number of patients: 75% (*n* = 47). SFI was associated with gender (*P* ≤ 0.05), age (*P* < 0.01), BMI (*P* < 0.01), chronic renal failure (*P* < 0.05), asthma (*P* < 0.05), and active cancer (*P* < 0.05; **Table 4**). SMI and SMD were assessed using thresholds defined by Martin et al. (15). A low SMI was present in 62% (*n* = 39) of patients, and a low SMD in 81% (*n* = 51). Low SMI was associated with BMI (*P* < 0.01) and 30-d mortality (*P* < 0.05; **Table 5**). A low SMD was associated with age (*P* < 0.05; **Table 6**).

**TABLE 3:** Clinicopathological characteristics and clinical outcomes in patients with COVID-19 as stratified by VFA^1^

Clinicopathological characteristic	All, *n* = 63	Low VFA, *n* = 21 (33.3%)	High VFA,^2^*n* = 42 (66.7%)	*P* value^3^
Sex				0.285
Male	30 (47.6)	12 (57.1)	18 (42.9)	
Female	33 (52.4)	9 (42.9)	24 (57.1)	
Age, y				0.571
<70	21 (33.3)	6 (28.6)	15 (35.7)	
≥70	42 (66.7)	15 (71.4)	27 (64.3)	
Ethnicity				0.715
White	59 (93.7)	20 (95.2)	39 (92.9)	
Other	4 (6.3)	1 (4.8)	3 (7.1)	
BMI, kg/m^2^				0.003
25–29	16 (51.6)	3 (14.3)	13 (31.0)	
≥30	15 (48.4)	1 (4.8)	14 (33.3)	
Smoking status				0.009
Current	10 (15.9)	7 (33.3)	3 (7.1)	
Ex	28 (44.4)	10 (47.6)	18 (42.9)	
Never	25 (39.7)	4 (19.0)	21 (50.0)	
Alcohol excess Hx.				0.241
Yes	11 (17.5)	2 (9.5)	9 (21.4)	
No	52 (82.5)	19 (90.5)	33 (78.6)	
Clinical frailty				0.356
Yes	45 (71.4)	17 (81.0)	28 (70.0)	
No	16 (25.4)	4 (19.0)	12 (30.0)	
Liver disease				
Yes	6 (9.5)	1 (4.8)	5 (11.9)	
No	57 (90.5)	20 (95.2)	37 (88.1)	0.363
Hypertension				0.721
Yes	34 (53.1)	12 (57.1)	22 (52.4)	
No	29 (45.3)	9 (42.9)	20 (47.6)	
Heart failure				0.539
Yes	8 (12.7)	2 (9.5)	6 (14.3)	
No	55 (87.3)	19 (90.5)	36 (85.7)	
T2DM				0.076
Yes	18 (28.1)	3 (14.3)	15 (35.7)	
No	45 (70.3)	18 (85.7)	27 (64.3)	
Chronic renal failure				0.348
Yes	11 (17.5)	5 (23.8)	6 (14.3)	
No	52 (82.5)	16 (76.2)	36 (85.7)	
Asthma				0.123
Yes	13 (20.6)	2 (9.5)	11 (26.2)	
No	50 (79.4)	19 (90.5)	31 (73.8)	
COPD				0.391
Yes	14 (22.2)	6 (28.6)	8 (19.0)	
No	49 (77.8)	15 (71.4)	34 (81.0)	
Active cancer				0.019
Yes	11 (17.5)	7 (33.3)	4 (9.5)	
No	52 (82.5)	14 (66.7)	38 (90.5)	
CRP, mg/L				0.188
≥10	52 (82.5)	6 (28.6)	21 (42.9)	
≥80	31 (49.2)	7 (33.3)	7 (22.2)	
≥150	14 (22.2)	8 (38.1)	14 (33.3)	
Albumin, g/L				0.271
<25	13 (20.6)	6 (28.6)	7 (16.7)	
≥25	50 (79.4)	15 (71.4)	35 (83.3)	
NLR				0.132
<3	10 (15.6)	1 (4.8)	9 (21.4)	
3–5	12 (18.8)	3 (14.3)	9 (21.4)	
>5	41 (64.1)	17 (81.0)	24 (57.1)	
poGPS				0.350
0	41 (65.1)	12 (57.1)	29 (69.0)	
1–2	22 (34.9)	9 (42.9)	13 (31.0)	
ITU admission				0.012
Yes	3 (4.8)	3 (14.3)	0 (0)	
No	60 (95.2)	18 (85.7)	42 (100)	
30-d mortality				0.002
Yes	11 (17.5)	8 (38.1)	3 (7.1)	
No	52 (82.5)	13 (61.9)	39 (92.9)	

^1^Values are *n* (%). COPD, chronic obstructive pulmonary disease; COVID-19, coronavirus disease; CRP, C-reactive protein; Hx., history; ITU, intensive therapy or care unit; NLR, neutrophil:lymphocyte ratio; poGPS, perioperative Glasgow Prognostic Score; T2DM, type 2 diabetes mellitus; VFA, visceral fat area.

2 High VFA defined as >160 cm^2^ for males and >80 cm^2^ for females.

3 *P* value is from χ^2^ analysis.

**TABLE 4:** Clinicopathological characteristics and clinical outcomes in patients with COVID-19 as stratified by SFI^1^

Clinicopathological characteristic	All, *n* = 63	Low SFI, *n* = 16 (25.4%)	High SFI,^2^*n* = 47 (74.6%)	*P* value^3^
Sex				0.050
Male	30 (47.6)	11 (68.8)	19 (40.4)	
Female	33 (52.4)	5 (31.3)	28 (59.6)	
Age, y				0.008
<70	21 (33.3)	1 (6.2)	20 (42.6)	
≥70	42 (66.7)	15 (93.8)	27 (57.4)	
Ethnicity				0.228
White	59 (93.7)	16 (100)	43 (91.5)	
Other	4 (6.3)	0 (0)	4 (8.5)	
BMI, kg/m^2^				0.002
25–29	16 (51.6)	2 (12.5)	14 (29.8)	
≥30	15 (48.4)	0 (0)	15 (31.9)	
Smoking status				0.113
Current	10 (15.9)	5 (31.3)	5 (10.6)	
Ex	28 (44.4)	7 (43.8)	21 (44.7)	
Never	25 (39.7)	4 (25.0)	21 (44.7)	
Alcohol excess Hx.				0.171
Yes	11 (17.5)	1 (6.3)	10 (21.3)	
No	52 (82.5)	15 (93.8)	37 (78.7)	
Clinical frailty				0.146
Yes	45 (71.4)	14 (87.5)	31 (68.9)	
No	16 (25.4)	2 (12.5)	14 (31.1)	
Liver disease				0.133
Yes	6 (9.5)	0 (0)	6 (12.8)	
No	57 (90.5)	16 (100)	41 (87.2)	
Hypertension				0.832
Yes	34 (53.1)	9 (56.3)	25 (53.2)	
No	29 (45.3)	7 (43.8)	22 (46.8)	
Heart failure				0.087
Yes	8 (12.7)	4 (25.0)	4 (8.5)	
No	55 (87.3)	12 (75.0)	43 (91.5)	
T2DM				0.314
Yes	18 (28.1)	3 (18.8)	15 (31.9)	
No	45 (70.3)	13 (81.3)	32 (68.1)	
Chronic renal failure				0.014
Yes	11 (17.5)	6 (37.5)	5 (10.6)	
No	52 (82.5)	10 (62.5)	42 (89.4)	
Asthma				0.018
Yes	13 (20.6)	0 (0)	13 (27.7)	
No	50 (79.4)	16 (100)	34 (72.3)	
COPD				0.757
Yes	14 (22.2)	4 (25.0)	10 (21.3)	
No	49 (77.8)	12 (75.0)	37 (78.7)	
Active cancer				0.014
Yes	11 (17.5)	6 (37.5)	5 (10.6)	
No	52 (82.5)	10 (62.5)	42 (89.4)	
CRP, mg/L				0.498
≥10	52 (82.5)	7 (43.8)	15 (38.5)	
≥80	31 (49.2)	5 (31.3)	10 (25.6)	
≥150	14 (22.2)	4 (25.0)	14 (35.9)	
Albumin, g/L				0.829
<25	13 (20.6)	3 (18.8)	10 (21.3)	
≥25	50 (79.4)	13 (81.3)	37 (78.7)	
NLR				0.905
<3	10 (15.6)	2 (12.5)	8 (17.0)	
3–5	12 (18.8)	3 (18.8)	9 (19.1)	
>5	41 (64.1)	11 (68.8)	30 (63.8)	
poGPS				0.116
0	41 (65.1)	13 (81.3)	28 (59.6)	
1–2	22 (34.9)	3 (18.8)	19 (40.4)	
ITU admission				0.746
Yes	3 (4.8)	1 (6.2)	2 (4.3)	
No	60 (95.2)	15 (93.8)	45 (95.7)	
30-dmortality				0.093
Yes	11 (17.5)	5 (31.3)	6 (12.8)	
No	52 (82.5)	11 (68.8)	41 (87.2)	

^1^Values are *n* (%). COPD, chronic obstructive pulmonary disease; COVID-19, coronavirus disease; CRP, C-reactive protein; Hx., history; ITU, intensive therapy or care unit; NLR, neutrophil:lymphocyte ratio; poGPS, perioperative Glasgow Prognostic Score; SFI, subcutaneous fat index; T2DM, type 2 diabetes mellitus.

2 High SFI defined as >50.0 cm^2^/m^2^ for males and >42.0 cm^2^/m^2^ for females.

3 *P* value is from χ^2^ analysis.

**TABLE 5:** Clinicopathological characteristics and clinical outcomes in patients with COVID-19 as stratified by SMI^1^

Clinicopathological characteristic	All, *n* = 63	Normal/high SMI, *n* = 24 (38.1%)	Low SMI,^2^*n* = 39 (61.9%)	*P* value^3^
Sex				0.824
Male	30 (47.6)	11 (45.8)	19 (48.7)	
Female	33 (52.4)	13 (54.2)	20 (51.3)	
Age, y				0.271
<70	21 (33.3)	10 (41.7)	11 (28.2)	
≥70	42 (66.7)	14 (58.3)	28 (71.8)	
Ethnicity				0.577
White	59 (93.7)	23 (95.8)	36 (92.3)	
Other	4 (6.3)	1 (4.2)	3 (7.7)	
BMI, kg/m^2^				0.003
25–29	16 (51.6)	3 (14.3)	13 (31.0)	
≥30	15 (48.4)	1 (4.8)	14 (33.3)	
Smoking status				0.182
Current	10 (15.9)	3 (12.5)	7 (17.9)	
Ex	28 (44.4)	8 (33.3)	20 (51.3)	
Never	25 (39.7)	13 (54.2)	12 (30.8)	
Alcohol excess Hx.				0.216
Yes	11 (17.5)	6 (25.0)	5 (12.8)	
No	52 (82.5)	18 (75.0)	34 (87.2)	
Clinical frailty				0.177
Yes	45 (71.4)	14 (63.6)	31 (79.5)	
No	16 (25.4)	8 (36.4)	8 (20.5)	
Liver disease				0.130
Yes	6 (9.5)	4 (16.7)	2 (5.1)	
No	57 (90.5)	20 (83.3)	37 (94.9)	
Hypertension				0.980
Yes	34 (53.1)	13 (54.2)	21 (53.8)	
No	29 (45.3)	11 (45.8)	18 (46.2)	
Heart failure				0.128
Yes	8 (12.7)	5 (20.8)	3 (7.7)	
No	55 (87.3)	19 (79.2)	36 (92.3)	
T2DM				0.623
Yes	18 (28.1)	6 (25.0)	12 (30.8)	
No	45 (70.3)	18 (75.0)	27 (69.2)	
Chronic renal failure				0.216
Yes	11 (17.5)	6 (25.0)	11 (17.5)	
No	52 (82.5)	18 (75.0)	52 (82.5)	
Asthma				0.976
Yes	13 (20.6)	5 (20.8)	8 (20.5)	
No	50 (79.4)	19 (79.2)	31 (79.5)	
COPD				0.677
Yes	14 (22.2)	6 (25.0)	8 (20.5)	
No	49 (77.8)	18 (75.0)	31 (79.5)	
Active cancer				0.896
Yes	11 (17.5)	4 (16.7)	7 (17.9)	
No	52 (82.5)	20 (83.3)	32 (82.1)	
CRP, mg/L				0.598
≥10	52 (82.5)	12 (50.0)	15 (38.5)	
≥80	31 (49.2)	4 (16.7)	10 (25.6)	
≥150	14 (22.2)	8 (33.3)	14 (35.9)	
Albumin, g/L				0.541
<25	13 (20.6)	4 (16.7)	9 (23.1)	
≥25	50 (79.4)	20 (83.3)	30 (76.9)	
NLR				0.245
<3	10 (15.6)	6 (25.0	4 (10.3)	
3–5	12 (18.8)	5 (20.8)	7 (17.9)	
>5	41 (64.1)	13 (54.2)	28 (68.3)	
poGPS				0.452
0	41 (65.1)	7 (29.2)	15 (38.5)	
1–2	22 (34.9)	17 (70.8)	24 (61.5)	
ITU admission				0.862
Yes	3 (4.8)	1 (4.2)	2 (5.1)	
No	60 (95.2)	23 (95.8)	37 (94.9)	
30-d mortality				0.029
Yes	11 (17.5)	1 (4.2)	10 (25.6)	
No	52 (82.5)	23 (95.8)	29 (74.4)	

^1^Values are *n* (%). COPD, chronic obstructive pulmonary disease; COVID-19, coronavirus disease; CRP, C-reactive protein; Hx., history; ITU, intensive therapy or care unit; NLR, neutrophil:lymphocyte ratio; poGPS, perioperative Glasgow Prognostic Score; SMI, skeletal muscle index; T2DM, type 2 diabetes mellitus.

2 Low SMI defined as BMI <25 kg/m^2^ and SMI <43 cm^2^/m^2^, or BMI ≥25 and SMI <53 cm^2^/m^2^ for males; and BMI <25 and SMI <41 cm^2^/m^2^, or BMI ≥25 and SMI <41 cm^2^/m^2^ for females.

3 *P* value is from χ^2^ analysis.

**TABLE 6:** Clinicopathological characteristics and clinical outcomes in patients with COVID-19 as stratified by SMD^1^

Clinicopathological characteristic	All, *n* = 63	Normal/high SMD, *n* = 12 (19.0%)	Low SMD,^2^*n* = 51 (81.0%)	*P* value^3^
Sex				0.035
Male	30 (47.6)	9 (75.0)	21 (41.2)	
Female	33 (52.4)	3 (25.0)	30 (58.8)	
Age, y				0.173
<70	21 (33.3)	6 (50.0)	15 (29.4)	
≥70	42 (66.7)	6 (50.0)	36 (70.6)	
Ethnicity				0.316
White	59 (93.7)	12 (100)	47 (92.2)	
Other	4 (6.3)	0 (0)	4 (7.8)	
BMI, kg/m^2^				<0.001
25–29	16 (51.6)	10 (83.3)	6 (11.8)	
≥30	15 (48.4)	2 (16.7)	13 (25.5)	
Smoking status				0.878
Current	10 (15.9)	2 (16.7)	8 (15.7)	
Ex	28 (44.4)	6 (50.0)	22 (43.1)	
Never	25 (39.7)	4 (33.3)	21 (41.2)	
Alcohol excess Hx.				0.107
Yes	11 (17.5)	4 (33.3)	7 (13.7)	
No	52 (82.5)	8 (66.7)	44 (86.3)	
Clinical frailty				0.175
Yes	45 (71.4)	7 (58.3)	38 (77.6)	
No	16 (25.4)	5 (41.7)	11 (22.4)	
Liver disease				0.876
Yes	6 (9.5)	1 (8.3)	5 (9.8)	
No	57 (90.5)	11 (91.7)	46 (90.2)	
Hypertension				0.759
Yes	34 (53.1)	6 (50.0)	28 (54.9)	
No	29 (45.3)	6 (50.0)	23 (45.1)	
Heart failure				0.614
Yes	8 (12.7)	1 (8.3)	7 (13.7)	
No	55 (87.3)	11 (91.7)	44 (86.3)	
T2DM				0.685
Yes	18 (28.1)	4 (33.3)	14 (27.5)	
No	45 (70.3)	8 (66.7)	37 (72.5)	
Chronic renal failure				0.355
Yes	11 (17.5)	1 (8.3)	10 (19.6)	
No	52 (82.5)	11 (91.7)	41 (80.4)	
Asthma				0.242
Yes	13 (20.6)	1 (8.3)	12 (23.5)	
No	50 (79.4)	11 (91.7)	39 (76.5)	
COPD				0.607
Yes	14 (22.2)	2 (16.7)	12 (23.5)	
No	49 (77.8)	10 (83.3)	39 (76.5)	
Active cancer				0.355
Yes	11 (17.5)	1 (8.3)	10 (19.6)	
No	52 (82.5)	11 (91.7)	41 (80.4)	
CRP, mg/L				0.817
≥10	52 (82.5)	5 (41.7)	22 (43.1)	
≥80	31 (49.2)	2 (16.7)	12 (23.5)	
≥150	14 (22.2)	5 (41.7)	17 (33.3)	
Albumin, g/L				0.242
<25	13 (20.6)	1 (8.3)	12 (23.5)	
≥25	50 (79.4)	11 (91.7)	39 (76.5)	
NLR				0.456
<3	10 (15.6)	3 (25.0)	7 (13.7)	
3–5	12 (18.8)	3 (25.0)	9 (17.6)	
>5	41 (64.1)	6 (50.0)	35 (68.6)	
poGPS				0.898
0	41 (65.1)	8 (66.7)	18 (35.3)	
1–2	22 (34.9)	4 (33.3)	33 (64.7)	
ITU admission				0.518
Yes	3 (4.8)	1 (8.3)	2 (3.9)	
No	60 (95.2)	11 (91.7)	49 (96.1)	
30-dmortality				0.355
Yes	11 (17.5)	1 (8.3)	11 (17.5)	
No	52 (82.5)	11 (91.7)	52 (82.5)	

^1^Values are *n* (%). COPD, chronic obstructive pulmonary disease; COVID-19, coronavirus disease; CRP, C-reactive protein; HU, Hounsfield unit; Hx., history; ITU, intensive therapy or care unit; NLR, neutrophil:lymphocyte ratio; poGPS, perioperative Glasgow Prognostic Score; SMD, skeletal muscle radiodensity; T2DM, type 2 diabetes mellitus.

2 Low SMD defined as BMI <25 kg/m^2^ and SMD <41 HU, or BMI ≥25 and SMD <33 HU for both sexes.

3 *P* value is from χ^2^ analysis.

Of the patients included, 3 (5%) had an ITU admission. Two patients were admitted directly to ITU from the ED, with 1 requiring escalation to a level 3 bed from ward-level care during admission. ITU admission was significantly associated with a high VFA (*P* < 0.05; Table 3). Thirty-day mortality was associated with high VFA (*P* < 0.05) and low SMI (*P* < 0.05; see Tables 3 and 5, respectively).

## Discussion

To our knowledge, this is the first study to explore the relation between CT-BC measurements, systemic inflammation, and outcomes in patients with COVID-19. The patients included were mainly elderly, were of white ethnicity, were systemically inflamed, overweight with subcutaneous and visceral obesity, and had sarcopenia using standard thresholds. Furthermore, sarcopenia in the presence of obesity was associated with clinical outcomes including greater 30-d mortality. Therefore, it would appear that body composition could have an important role in predicting clinical outcome in patients presenting with COVID-19. Further large-scale studies are warranted to establish the prognostic role of body composition in these patients.

Numerous studies have suggested that obesity, as measured by BMI, is associated with poorer outcomes in patients with COVID-19 (5, 17). However, BMI reflects both fat and muscle mass in the body and therefore it is not clear whether such increased risk is due to high fat mass, low muscle mass, or both. In the present study visceral obesity appeared to be associated with a lower 30-d mortality whereas sarcopenia was associated with a higher 30-d mortality. The basis of this divergence of body composition components and clinical outcome is not clear. However, a low muscle mass against a background of an acute (18) or chronic inflammatory state has long been recognized to be associated with poor clinical outcomes (19). Irrespective, it will be important to carry out further body composition studies in patients with COVID-19 .

Sarcopenia has been shown to be prevalent in the elderly population as well as those with cancer (20, 21). The prevalence of a low SMI in this COVID-19 cohort was ∼50% when those with cancer were excluded. If we compare this with cohorts of patients with curative colorectal and advanced lung cancer, similar levels of prevalence of a low SMI are observed (8, 9). This would suggest that sarcopenia is endemic and not exclusive to those with COVID-19. This brings into question of how to mitigate the inflammatory effects of COVID-19 in such patients. Clearly, moderation of the systemic inflammatory response could be important, and indeed randomized controlled trials have shown the value of anti-inflammatory agents (22). From the present results it can be speculated that COVID-19 patients with sarcopenia will benefit most from such therapeutic agents.

Systemic inflammation has been shown to be associated with poor outcomes in patients with COVID-19 (23). In addition, several studies have shown the negative impact of an elevated NLR on those with COVID-19 (24, 25). The poGPS is a validated score that is independently associated with infective complications and 30-d mortality in patients undergoing surgery (12). This score was chosen due to the significant degree of inflammation exhibited by those with COVID-19. A similar prevalence of systemic inflammation, as measured by CRP ≥80, poGPS ≥1, and NLR to >5, was observed in the present cohort (49%, 36%, and 62%, respectively), and across the entire cohort from which the patients in this study were identified (51%, 25%, and 55%, respectively) (23). Furthermore, when compared with COVID-19 cohorts from further afield, such as the Far East, such systemic inflammation was also prevalent (26). Therefore, activation of the host systemic inflammatory response is a consistent feature of this disease. From the present results it may be speculated that the prognostic value and treatment of the systemic inflammatory response will be greatest in those COVID-19 patients with sarcopenia.

There are a number of limitations of this present study. Importantly, this study is a single-center study with a small sample size and therefore subject to sample bias. Although the present study has a small sample size, it is important to highlight that not all patients with COVID-19 undergo routine CT imaging in the United Kingdom. Within the literature there is a single study with a larger cohort than ours (27). However, they used a nonstandardized methodology for the calculation of SMI (27). Two other smaller studies assessed the relation between VFA and clinical outcomes in those with COVID-19 (6, 7). To our knowledge, the present study has the largest cohort to date exploring the relation between CT body composition measurements, systemic inflammation, and clinical outcomes in patients with COVID-19. Therefore, the present cohort provides a novel insight into the relation of body composition and systemic inflammation in those with COVID-19. Furthermore, although it is possible that the relation of SMI with mortality was an age-related factor, when patients older than 65 y were excluded from the univariate analysis, the association between SMI and 30-d mortality remained significant (*n* = 21, *P* = 0.028). A larger cohort of patients will be required to address this point in detail.

In summary, sarcopenia in the presence of obesity was associated with clinical outcomes including greater 30-d mortality. Therefore, it would appear that body composition can have an important role in predicting clinical outcome in patients presenting with COVID-19.

## Data Availability

Data described in the manuscript will be made available upon request pending application and approval of the senior author.

## Notes

The authors reported no funding received for this study.

Author disclosures: The authors report no conflicts of interest.

Abbreviations used: AAU, Acute Assessment Unit; COVID-19, coronavirus disease; CRP, C-reactive protein; CT, computed tomography; CT-BC, CT-derived body composition; ED, Emergency Department; GRI, Glasgow Royal Infirmary; ITU, intensive therapy or care unit; NHS, National Health Service (UK); NLR, neutrophil:lymphocyte ratio; poGPS, perioperative Glasgow Prognostic Score; SFI, subcutaneous fat index; SMA, skeletal muscle area; SMD, skeletal muscle radiodensity; SMI, skeletal muscle index; TFA, total fat area; VFA, visceral fat area.
